# ScWRKY6 Interacts With ScSAG39 to Regulate Immune Homeostasis by Transcriptional Control of *ScPR1*

**DOI:** 10.1111/pbi.70444

**Published:** 2025-11-07

**Authors:** Shoujian Zang, Dongjiao Wang, Liqian Qin, Shijiang Cui, Guran Wu, Kaisheng Liu, Qiugang Ding, Qianlong Hui, Tingting Sun, Yachun Su, Yingfang Zhu, Qibin Wu, Youxiong Que

**Affiliations:** ^1^ State Key Laboratory of Tropical Crop Breeding, Institute of Tropical Bioscience and Biotechnology/Sanya Research Institute Chinese Academy of Tropical Agricultural Sciences Sanya Hainan China; ^2^ Key Laboratory of Sugarcane Biology and Genetic Breeding, Ministry of Agriculture and Rural Affairs, National Engineering Research Center for Sugarcane, Key Laboratory of Applied Genetics of Universities in Fujian Province, College of Agriculture Fujian Agriculture and Forestry University Fuzhou China; ^3^ Sanya Institute of Henan University Sanya Hainan China

**Keywords:** immune homeostasis, pathogenesis‐related gene, sugarcane, WRKY transcription factor

1

Sugarcane (*Saccharum* spp.) is essential for global sugar and bioenergy production, but its yield and quality are severely threatened by fungal diseases (Ling et al. 2025). Plant defence against pathogens is primarily regulated by transcription factors (TFs) (Buscaill and Rivas 2014), among which WRKYs can act as positive or negative immune regulators (Huang et al. 2022). We previously reported that ScWRKY4 interacts with ScJAZ13 to suppress JA signalling and immune gene expression, increasing susceptibility to pathogens (Wang et al. 2024). More recently, we found that ScWRKY2 reduces resistance to smut disease by interacting with the chloroplast protein ScPsbP and inducing ROS scavenging genes (Wang et al. 2025). These findings indicate that WRKY TFs play diverse roles in sugarcane immunity. However, their contribution to immune homeostasis during fungal infection remains unclear.

Here, we identified the sugarcane ScWRKY6, a class II‐d WRKY TF, which contains two nuclear localization signals (NLSs), two nuclear export signals (NESs), a conserved zinc finger motif, and a WRKY domain (Figure 1A,B; Figure S1A–C; Table S1). Its expression is markedly induced by smut, pokkah boeng, and brown stripe diseases, suggesting its potential role in the broad‐spectrum antifungal response of sugarcane (Figure 1C). Notably, ScWRKY6 was revealed as a nuclear protein that promotes intracellular ROS accumulation, as indicated by elevated ROS‐scavenging gene expression and stronger H_2_DCF‐DA (2′, 7′‐dichlorodihydrofluorescein diacetate) fluorescence (Figure 1D,E; Figure S1D). These results suggest that ScWRKY6 may function as a regulator in sugarcane response to fungal pathogens.

**FIGURE 1:**
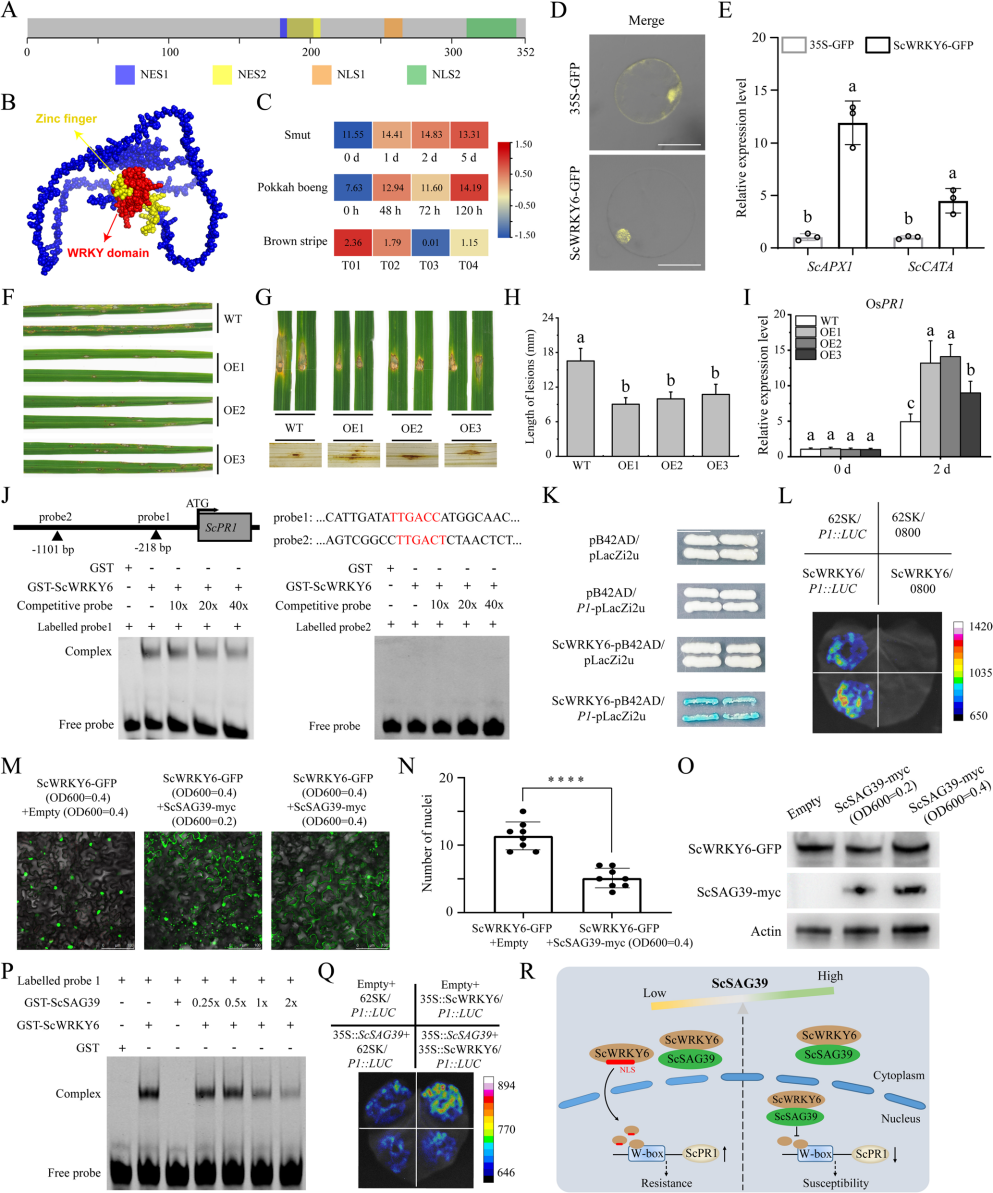
ScWRKY6‐ScSAG39 module regulates *ScPR1* expression and immune homeostasis. (A) Conserved domains of the ScWRKY6 protein and its predicted NLS and NES. (B) 3D structural diagram of the ScWRKY6 protein. (C) Expression patterns of the *ScWRKY6* gene in response to smut, pokkah boeng, and brown stripe diseases. (D) Subcellular localization of the ScWRKY6‐GFP fusion protein in sugarcane protoplasts. (E) The expression levels of genes involved in ROS scavenging after protoplast transfection (Student's *t*‐test, *p* < 0.01). (F) Determination of the rice blast resistance of the *ScWRKY6*‐overexpression lines via the spray method. (G) Disease response and H_2_O_2_ accumulation in WT and *ScWRKY6‐*overexpression plants. (H) Statistical analysis of the spread length of diseased spots in WT and *ScWRKY6*‐overexpression plants (Student's *t*‐test, *p* < 0.05, *n* = 3). (I) The expression level of Os*PR1* in WT and *ScWRKY6*‐overexpression plants before and after *M. oryzae* infection. Different letters indicate significant difference (Student's *t*‐test, *p* < 0.05, *n* = 3). (J) WRKY domain of ScWRKY6 directly bound to probe 1 but not probe 2 of the W‐box elements in EMSA assays. (K) ScWRKY6 directly associated with the *P1* fragment of *ScPR1* promoter in yeast one‐hybrid assays. (L) Dual‐luciferase expression assays showing the transcriptional activation of *ScPR1* promoter activity by ScWRKY6. (M) Effects of different concentrations of ScSAG39‐myc on the subcellular localization of ScWRKY6‐GFP. (N) Quantification of nuclear number in ScWRKY6‐GFP‐expressing cells with different concentrations of ScSAG39‐myc (Student's *t*‐test, ****p < 0.0001). (O) Determination of protein levels after co‐infiltration of ScSAG39‐myc at different concentrations with ScWRKY6‐GFP. (P) The effect of the ScSAG39 protein on the binding ability of ScWRKY6 with *ScPR1* promoter in the EMSA assays. (Q) *ScSAG39* repressed the ScWRKY6‐mediated activation of *ScPR1* transcription. (R) This model illustrates the mechanism by which ScWRKY6 interacts with ScSAG39 to regulate immune responses.

Subsequently, a rice ( *Oryza sativa* )–*Magnaporthe oryzae* system was established to investigate the regulatory role of ScWRKY6 in fungal disease resistance using genetic and biochemical approaches (Figure S1E–G). Interestingly, *ScWRKY6*‐OE transgenic plants exhibited fewer disease lesions and significantly higher H_2_O_2_ accumulation than wild‐type (WT) plants after inoculation with the *M. oryzae* strain Guy11 (Figure 1F,G). In punch inoculation assays, lesion lengths in *ScWRKY6*‐OE plants were significantly shorter than those in WT (Figure 1G,H). Besides, the expression level of the defence‐related gene *OsPR1* was markedly higher in *ScWRKY6*‐OE plants at 48 h post‐inoculation (Figure 1I), indicating that ScWRKY6 could positively regulate rice resistance to *M. oryzae* .

To elucidate the role of ScWRKY6 in rice blast resistance, we performed RNA‐seq on 12 cDNA libraries from WT and *ScWRKY6*‐OE1 plants inoculated with (T) or without *M. oryzae* (CK) (Figure S2A; Table S2). A total of 4947 and 1838 DEGs were identified in the WT‐CK_vs_WT‐T and ScWRKY6‐CK_vs_ScWRKY6‐T groups, respectively (Figure S2B–E). Among them, the up‐regulated genes in WT‐CK_vs_WT‐T were primarily associated with fundamental metabolic and biosynthetic pathways. In contrast, the up‐regulated genes in ScWRKY6‐CK_vs_ScWRKY6‐T were predominantly enriched in pathways related to plant immune responses (Figure S2F,G; Table S3). Interestingly, *ScWRKY6* overexpression activates the phenylpropanoid biosynthesis pathway, with most related DEGs up‐regulated, including key enzymes like PAL, C4H, and 4CL (Figure S3). Surprisingly, but reasonably, numerous TFs and resistance (*R*) genes, such as *NLR‐*, *RLK‐* and *WAK‐type* genes, were specifically up‐regulated in *ScWRKY6*‐OE lines (Figure S4; Tables S4 and S5), indicating that ScWRKY6 enhances rice defence against *M. oryzae* by promoting secondary metabolism and *R* gene expression.

Furthermore, we showed that ScWRKY6 could up‐regulate the expression of *OsPR1* to mediate plant immune responses (Figure 1I). To determine whether it directly regulates *PR1*, the promoter of the homologous gene *ScPR1*, which was transcriptionally active and inducible upon *Sporisorium scitamineum* infection, was cloned from sugarcane (Figure S5A–C). This promoter contained two W‐box motifs, one of which (probe1) was specifically bound by ScWRKY6 (Figure 1J,K; Figure S5D). Dual‐luciferase and ChIP‐qPCR assays further demonstrated that ScWRKY6 could activate *ScPR1* transcription (Figure 1L; Figure S6). These results indicate that ScWRKY6 directly binds to the *ScPR1* promoter, most probably contributing to the pathogen‐induced expression of *ScPR1*.

To identify the potential ScWRKY6‐interacting proteins involved in disease responses, a yeast two‐hybrid (Y2H) screen was conducted using a sugarcane cDNA library from smut‐infected buds (Figure S7A). They were mainly involved in arabinose metabolism, protein degradation, and cysteine protease activity (Figure S7B). Among them, *ScSAG39*, a cysteine protease gene down‐regulated after *S. scitamineum* infection (Figure S8A–C), was selected for further study. Overexpression of *ScSAG39* in *Nicotiana benthamiana* aggravated disease symptoms and reduced H_2_O_2_ accumulation, implying a negative role in plant defence. This was further supported by altered expression of ROS‐ and HR‐related genes (Figure S8D–F).

We also found that ScSAG39 was an endoplasmic reticulum–associated protein (Figure S8G). Its interaction with ScWRKY6 was confirmed by Y2H, BiFC, and Co‐IP assays (Figure S9). Notably, co‐expression assays showed that changes in ScSAG39 abundance altered the nuclear localization of ScWRKY6 (Figure 1M–O). ScSAG39 restricted the nuclear import of ScWRKY6, and reduced ScSAG39 levels allowed more ScWRKY6 to accumulate in the nucleus (Figure 1N; Figure S9A). In addition, ScSAG39 also inhibited the activation of *ScPR1* by competitively blocking ScWRKY6 binding to the W‐box element (Figure 1P), and its co‐expression with ScWRKY6 led to a marked decrease in luciferase activity driven by the *ScPR1* promoter (Figure 1Q; Figure S10). Furthermore, both ScSAG39 and the W‐box element interacted with the same key residue, lysine 310 of ScWRKY6 (Figure S11), which informed us that ScSAG39 competitively occupied the site required for transcriptional activation of *ScPR1*.

Overall, this study demonstrates the positive role of sugarcane ScWRKY6 in enhancing fungal disease resistance. ScWRKY6 interacts with ScSAG39. Reduced ScSAG39 levels lead to greater nuclear accumulation of ScWRKY6 and activation of *ScPR1*, whereas high ScSAG39 levels inhibit *ScPR1* expression by interfering with ScWRKY6 binding to the W‐box element, thereby modulating plant immune responses (Figure 1R). These findings provide novel insights into the regulation of plant immune homeostasis.

## Author Contributions

Y.Q., Q.W., and Y.Z. conceived and designed the project. S.Z., D.W., L.Q., S.C., and Q.H. analyzed the data. S.Z., G.W., K.L., Q.D., and T.S. performed the experiments. S.Z. and Q.W. wrote the manuscript draft. Y.Q., Q.W., Y.Z., and Y.S. revised the manuscript.

## Supporting information


**Tables S1–S6.** pbi70444‐sup‐0001‐TableS1–S6.xlsx.

**Figures S1–S11.** pbi70444‐sup‐0002‐FigureS1–S11.docx.

## Data Availability

The RNA‐seq data have been deposited at Beijing Institute of Genomics Data Center (http://bigd.big.ac.cn), accession number is PRJCA036085.

## References

[pbi70444-bib-0001] Buscaill, P. , and S. Rivas . 2014. “Transcriptional Control of Plant Defence Responses.” Current Opinion in Plant Biology 20: 35–46. 24840291 10.1016/j.pbi.2014.04.004

[pbi70444-bib-0002] Huang, X. , L. Cao , J. Fan , G. Ma , and L. Chen . 2022. “CdWRKY2‐Mediated Sucrose Biosynthesis and CBF‐Signalling Pathways Coordinately Contribute to Cold Tolerance in Bermudagrass.” Plant Biotechnology Journal 20: 660–675. 34743386 10.1111/pbi.13745 PMC8989505

[pbi70444-bib-0003] Ling, H. , X. Fu , N. Huang , et al. 2025. “A Sugarcane Smut Fungus Effector Hijacks Plant Vacuolar Sorting Receptor‐Mediated Trafficking to Evade Host Immune Detection.” Plant, Cell & Environment 48: 5271–5289. 10.1111/pce.15500 PMC12131962 40166905

[pbi70444-bib-0004] Wang, D. , Y. Gou , C. Yi , et al. 2025. “ScWRKY2: A Key Regulator for Smut Resistance in Sugarcane.” Plant Biotechnology Journal 23: 3667–3681. 10.1111/pbi.70186. 40488671 PMC12392974

[pbi70444-bib-0005] Wang, D. , W. Wang , S. Zang , et al. 2024. “Sugarcane Transcription Factor ScWRKY4 Negatively Regulates Resistance to Pathogen Infection Through the JA Signaling Pathway.” Crop Journal 12: 164–176.

