# Supplementary material for: ScWRKY6 Interacts With ScSAG39 to Regulate Immune Homeostasis by Transcriptional Control of ScPR1
Source: Plant Biotechnol J. 2025 Nov 7;24(3):1766–8. doi: 10.1111/pbi.70444 (PMC12946490; doi:10.1111/pbi.70444)
Supplement: Supplementary file 2 — Figures S1–S11. pbi70444‐sup‐0002‐FigureS1–S11.docx. [file PBI-24-1766-s001.docx]

# Supplemental materials and methods

## Plant materials and treatments

Sugarcane cultivar ROC22 was provided by the Key Laboratory of Sugarcane Biology and Genetic Breeding, Ministry of Agriculture and Rural Affairs, Fujian Agriculture and Forestry University (FAFU), Fuzhou, Fujian. For the smut pathogen infection, mature stems of 10-month-old ROC22 sugarcane were selected, cut into single-bud segments, and cultured hydroponically in a 30 °C incubator with a 16-hour light/8-hour dark cycle. Once the sugarcane buds had grown to approximately 2.0 cm, they were inoculated with *Sporisorium scitamineum*. Bud samples were collected at 0 (control), 1, 3, and 5 days post-inoculation (dpi), with three biological replicates, each containing five buds (Sun et al., 2023). The rice plants utilized comprised wild-type (WT) Nipponbare and transgenic lines overexpressing *ScWRKY6* gene (GeneBank accession no. MH393927). *ScWRKY6* transgenic rice plants were obtained by infecting rice callus with *Agrobacterium tumefaciens* carry the pBWA(V)BU-ScWRKY6 plasmid (without tag). PCR detection was then performed using the primer pair G-*ScWRKY6* to identify the positive transgenic plants (Supplementary Table S6). Three T_3_ transgenic homozygous lines overexpressing *ScWRKY6* (*ScWRKY6*-OE1, *ScWRKY6*-OE2, and *ScWRKY6*-OE3) were randomly selected for resistance analysis. All rice plants were cultivated either in the paddy fields at FAFU, or in a greenhouse with a 16-hour light/8-hour dark cycle at 28 °C and 70% relative humidity.

## RNA extraction and RT-qPCR assay

Total RNA was isolated using TRIzol reagent (Yeasen, Shanghai, China), and cDNA was synthesized with the Hifair III 1st Strand cDNA Synthesis SuperMix kit (Yeasen, Shanghai, China). Real-time quantitative PCR (RT-qPCR) detection was performed using the ABI 7500 Real-Time PCR System (Thermo, Waltham, USA), following the protocol provided with the SYBR Green PCR Master Mix Kit. The expression level of ScWRKY6 was quantified using specific primers ScWRKY6-Q, with glyceraldehyde-3-phosphate dehydrogenase (GAPDH) serving as the internal reference gene for sugarcane (Ling et al., 2014). For rice, the ubiquitin gene was used as internal reference for normalization (Yang et al., 2012). Total DNA from infected leaves was extracted for RT-qPCR in fungal biomass assays. Quantifying relative fungal biomass by measuring the expression level of the *Pot2* gene in *Magnaporthe oryzae* (Lu et al., 2024)*.* The relative gene expression was calculated according to the 2^-ΔΔCT^ algorithm (Livak et al., 2001).

## Bioinformatics analysis of ScWRKY6

The TAIR database (<https://www.arabidopsis.org/>) was utilized to obtain the protein sequences of all WRKY and PLCP proteins in *Arabidopsis*, and evolutionary trees were constructed using the neighbor-joining (NJ) method with MEGA-X software (Kumar et al., 2018). Structural domains were identified using the Conserved Domains database (<http://www.ncbi.nlm.nih.gov/Structure/cdd/wrpsb.cgi>). The structural model of ScWRKY6 was builted based on AlphaFold 3 and the conserved domains were aligned and visualized using PyMOL3.1 (Abramson et al., 2024). Nuclear localization signal (NLS) and nuclear export signal (NES) motifs were predicted using the cNLS Mapper program (Kosugi et al., 2009) and the LocNES tool (Xu et al., 2015), respectively. Two thousand bp promoter sequences upstream of *ScWRKY6* homologous genes were retrieved, and cis-acting regulatory elements were predicted using the PlantCARE online tool (<https://bioinformatics.psb.ugent.be/webtools/plantcare/html/>), the results were then visualized using TBtools (Chen et al., 2023). Sequence alignment was performed using the ESPript web tool (<https://espript.ibcp.fr/ESPript/cgi-bin/ESPript.cgi>).

## Isolation and transient transfection of sugarcane protoplasts

Protoplasts were isolated as previously described (Zang et al., 2025). Briefly, protoplasts from sugarcane leaf sheath tissue were adjusted to 2 × 10⁶ cells/mL. For transformation, 4 μg of plasmid DNA was mixed with 100 μL of protoplast suspension, with an empty vector as the control. An equal volume of 40% (w/v) PEG was added, and the mixture was incubated in the dark for 15 minutes. Transformation was stopped by adding W5 solution, followed by centrifugation at 100 × g for 3 minutes. The protoplasts were then incubated at room temperature in the dark for 14-16 hours prior to RNA extraction and subcellular localization analysis (You et al., 2016). Following transient transfection, protoplasts were stained with the fluorescent probe 2′, 7′-dichlorodihydrofluorescein diacetate (H_2_DCF-DA) to assess ROS levels (Li et al., 2011). Stained protoplasts were then observed and imaged using a confocal laser scanning microscope (Leica TCS SP8, Wetzlar, Germany).

## Rice blast fungus inoculation assay

Inoculations were performed using the *M. oryzae* isolate Guy11 (Lu et al., 2024). Rice blast spores were washed and suspended in a 0.02% Tween-20 solution in rice bran medium, adjusted to a concentration of 10^5^ spores/mL. The spore suspension was sprayed onto the leaves of approximately 3-week-old healthy rice plants. Disease symptoms on the leaves were assessed and photographed at 7 days post-inoculation. For punch inoculation, a circular wound was gently created on one side of each leaf of four-week-old uniform rice plants using a punch. Subsequently, approximately 10 µL of spore suspension was inoculated onto the wounds and sealed with transparent tape. Lesion length was measured at 6 days after inoculation.

## RNA sequencing and data analysis

RNA-seq was used to analyze the transcriptome changes in WT and ScWRKY6-OE1 plants in response to *M. oryzae* attack (Zhang et al., 2024). For the transcriptome assembly, 12 cDNA libraries were constructed for WT and ScWRKY6-OE1 plants inoculated with rice blast fungus at 0 d and 7 d, respectively (Sun et al., 2023). The samples were designated as WT-CK, WT-T, ScWRKY6-CK, and ScWRKY6-T, respectively. Each type of sample included three biological replicates and was sequenced using the NovaSeq 6000 platform (Illumina Inc., CA, USA) by Genedenovo Biotechnology Co., Ltd. (Guangzhou, China). The raw reads were quality controlled using fastp to filter out low-quality data and mapped to the rice Nipponbare genome (Shang et al., 2023) using HISAT2 (Kim et al., 2015). Differential expression analysis was performed using DESeq2 with the default parameters (Fold Change > 2 and *P*-value < 0.05) to identify differentially expressed genes (DEGs) (Love et al., 2014). Venn diagram analysis, Gene Ontology (GO) enrichment, and Kyoto Encyclopedia of Genes and Genomes (KEGG) pathway enrichment were performed using the Venn draw tools, ClusterProfiler package, and ggplot2, respectively (Sun et al., 2023). To identify disease resistance-related *R* genes involved in pathogen responses, predictions were made with the PRGdb 4.0 software (Calle García et al., 2022). Moreover, TFs participating in the ScWRKY6-OE1-mediated response to pathogens were predicted using the PlantRegMap database (Tian et al., 2020). The RNA-seq data have been deposited at Beijing Institute of Genomics Data Center (<http://bigd.big.ac.cn>), accession number is PRJCA036085.

## Cloning and sequence analysis of gene and promoter

The *ScSAG39* gene (GenBank accession no. PV361547) and the *ScPR1* promoter (*proScPR1*) (GenBank accession no. PV361548) were cloned from the sugarcane cultivar ROC22 using specific primers (Supplementary Table S6). A phylogenetic tree of ScSAG39 was constructed with MEGA-X software, using *Arabidopsis* PLCP proteins as references (Zhang et al., 2019). To examine whether ScWRKY6 directly regulates PR1 in sugarcane, 19 previously reported *SsPR1* genes (Chu et al., 2022) were retrieved, and their expression patterns were analyzed in response to *Sporisorium scitamineum* infection in both the smut-susceptible cultivar ROC22 and the smut-resistant cultivar YC05-179. The promoter of the homologous gene *Sspon.02G0024690-2B* from ROC22, which was up-regulated upon *S. scitamineum* infection, was subsequently cloned and designated as *proScPR1*.

## Electrophoretic mobility shift assays (EMSA)

After codon optimization and synthesis, the WRKY domain of ScWRKY6 was cloned into the pGEX-4T-1 vector. The GST-tagged recombinant protein GST-WRKY6-domain was expressed in *E. coli* Rosetta using IPTG induction and then purified. DNA fragments were end-labeled with Cy5 (Table S6), and the fluorescence-labeled DNA was incubated with serially different concentrations of the purified protein in 5× binding buffer (Beyotime, Shanghai, China). For competition assays, serially increasing concentrations of non-labeled competitor DNA was added to the reaction. Electrophoresis of the reaction mixture was conducted on a 6% native polyacrylamide gel in 0.5× Tris-borate-EDTA buffer for 1 hour at 120 V, and the results were scanned with the Odyssey CLX imaging system (LI-COR, Lincoln, USA) (Liu et al., 2024).

## Yeast one-hybrid assay

The coding sequence of ScWRKY6 was cloned into the pB42AD vector, and the *P1* fragment of *proScPR1* (GeneBank accession no. PV361548), which contained the 100 bp sequences flanking probe1, was inserted into the pLacZi2u vector, and yielded the constructs: ScWRKY6-pB42AD and proScPR1-P1-pLacZi2u (primers were listed in Table S6). These fusion constructs were co-transformed into the yeast strain EGY48. After three days of growth on SD/-Trp/-Ura medium, the yeast was transferred to a medium containing raffinose, galactose, 10× BU buffer (0.25 M Na_2_HPO_4_·7H_2_O and 0.25 M NaH_2_PO_4_), and 4 mg/mL X-β-Gal (Coolaber, Beijing, China), and incubated at 30°C to assess their transcriptional activities.

## Dual-luciferase reporter (LUC) assay

The full-length coding sequence of ScWRKY6 was cloned into the pGreen II 62-SK vector, while the *P1* fragment of *proScPR1*, was inserted into the pGreen II 0800-LUC vector (primers were listed in Table S6). The recombinant vectors were transformed into the *A. tumefaciens* strain GV3101 and subsequently injected into tobacco leaves for transient co-transformation expression analysis. The leaves were then sprayed with a solution of D-luciferin potassium salt containing 0.1% Triton X-100, and luminescence was recorded using a low-light cooled charge-coupled device camera (PerkinElmer, USA). Firefly and Renilla luciferase activities were measured using a dual-luciferase reporter assay system (Beyotime, Shanghai, China) following the manufacturer’s instructions. All luminescence measurements and luciferase activity assays were conducted with three biological replicates.

## Chromatin immunoprecipitation (ChIP) assay

ChIP-qPCR was performed using the BeyoChIP™ ChIP Assay Kit (Beyotime, China, P2080S) according to the manufacturer’s protocol with minor modifications. Briefly, transformed protoplast samples were cross-linked at 37 °C with 1% (v/v) formaldehyde for 10 min. Cross-linking was quenched by adding 1.25 M glycine, followed by incubation for 5 min. The samples were then subjected to sonication to shear chromatin into fragments of approximately 200-700 bp. Ten percent of the sonicated chromatin was reserved as input control and stored at -80 °C, while the remaining chromatin was used for immunoprecipitation (IP). The sheared chromatin was incubated with magnetic beads conjugated to anti-GFP antibody for at least 2 h. DNA was then recovered from the beads and purified for subsequent ChIP-qPCR analysis. The enrichment fold of each fragment was normalized to the internal control, and then by normalizing the value for the IP samples against that for the input. All primers used for ChIP-qPCR are listed in Table S6.

## Yeast two-hybrid (Y2H) screen and interaction assay

The Y2H screening and methods used for the identification of ScWRKY6 followed the protocol provided by the manufacturer (Oebiotech, Shanghai, China). The full coding sequence of ScWRKY6 was fused with the pGBKT7 vector and transformed into the yeast strain Y2HGold as the bait construct. After testing for self-activation of ScWRKY6, the library plasmid was transformed into the yeast strain Y187, and a mating assay was conducted to screen the library (You et al., 2016). Positive clones were screened on SD/-His/-Leu/-Trp medium containing X-α-Gal and subsequently sequenced. For the Y2H assay, recombinant plasmids were co-transformed into chemically competent Y2HGold cells and selected on SD/-Leu/-Trp medium. The transformants were then further screened on SD/-Leu/-Trp/-His/-Ade medium to assess potential protein–protein interactions (Ling et al., 2022).

## Subcellular localization and bimolecular fluorescence complementation (BIFC) assay

During subcellular localization, the complete coding sequences of ScSAG39 (GeneBank accession no. PV361547), ScWRKY6^NLS^ (ScWRKY6 nuclear localization signal motif knockout sequence) and ScWRKY6^NES^ (ScWRKY6 nuclear export signal motif knockout sequence) were amplified using specific primers (Table S6) and subsequently cloned into the pCAMBIA1300-GFP vectors. All constructs were then transformed into *A.* *tumefaciens* strain GV3101 and injected into *Nicotiana benthamiana* leaves (Pan et al., 2016). GFP and RFP signals were assessed and photographed using a confocal laser scanner microscope (Leica TCS SP8, Wetzlar, Germany) at 48 h after injection. For the BIFC assay, the complete coding sequences of ScWRKY6, ScWRKY6^NLS^, ScWRKY6^NES^ and ScSAG39 were cloned using the Gateway method and inserted into the pEarleyGate 201 and pEarleyGate 202 vectors, respectively. These vectors carry the N-terminal or C-terminal of yellow fluorescent protein (YFP), respectively (Zang et al., 2025). Subsequently, proteins fused to the N-terminus and C-terminus of YFP were co-expressed in *N. benthamiana* leaves via *Agrobacterium*-mediated transient transformation, and the resulting YFP signals were visualized using a confocal laser scanner microscope.

## Co-immunoprecipitation (Co-IP) assay

In Co-IP assay, the coding sequences of ScWRKY6 and ScSAG39 were cloned into the PCAMBI1300-GFP vector and the pEarleyGate203 vector, respectively, to generate ScWRKY6-GFP and ScSAG39-myc fusion proteins. The recombinant constructs were then transformed into *A. tumefaciens* and used to inject tobacco leaves. The co-transformation of GFP empty vector and the ScSAG39-myc served as the negative control. After 48 hours, total protein was extracted using protein extraction buffer (Beyotime, Shanghai, China) and incubated with anti-GFP beads (AlpalifeBio, Shenzhen, China) at 4°C for 3 hours. The beads were washed five times with 1× PBS buffer, and the samples were then separated by SDS-PAGE and analyzed by western blotting using anti-GFP and anti-myc antibodies (Abmart, Shanghai, China).

## Transient overexpression of *ScSAG39* in *N. benthamiana*

*Agrobacterium* cultures carrying either *35S::ScSAG39* or the empty vector 35S::00 (as a control) were infiltrated into the leaves of healthy tobacco plants at the 6-7 leaf stage (Zang et al., 2022). After 1 day, the leaves were inoculated with the fungal pathogen *Alternaria alternata*. The expression levels of immune-related marker genes in the treated leaves were then analyzed using RT-qPCR. These genes included *NbCAT1* and *NbSOD*, which were associated with the ROS pathway, as well as *NbHSR201* and *NbHSR203*, involved in the HR pathway (Sun et al., 2023). All of the primers used for RT-qPCR were listed in Supplementary Table S6.

## DAB and GUS staining

To analyze H₂O₂ accumulation in rice leaves, WT and *ScWRKY6*-OE plants were grown in a greenhouse for three weeks and then sprayed with *M. oryzae*. After 48 hours, the leaves were collected, immersed in DAB staining solution, vacuum-infiltrated for 30 minutes, and incubated in darkness overnight. On the following day, the staining solution was replaced with absolute ethanol, and the leaves were boiled in 95% ethanol until fully decolorized. The DAB staining was employed to detect hydrogen peroxide (H_2_O_2_) in *N. benthamiana* leaves according to our previous studies (Sun et al., 2024). For GUS staining, the tobacco leaves with *Agrobacterium*-mediated transient transformation were incubated in GUS staining solutions, containing 80 mM sodium phosphate buffer, 0.5 mM potassium ferricyanide, 0.5 mM potassium ferrocyanide, 10 mM EDTA, 1 mg/mL 5-Bromo-4chloro-indolyl-β-D-glucuronide (X-Gluc), and 0.1% Triton X-100 for 24 h at 37 °C. After staining, the tissues were transferred to 70% ethanol to decolorize at 37 °C for about two days.

**Supplemental References**

Abramson, J., Adler, J., Dunger, J., Evans, R., Green, T., Pritzel, A., Ronneberger, O., Willmore, L., Ballard, A. J., Bambrick, J., et al. (2024). Accurate structure prediction of biomolecular interactions with AlphaFold 3. Nature 630:493–500.

Calle García, J., Guadagno, A., Paytuvi-Gallart, A., Saera-Vila, A., Amoroso, C. G., D'Esposito, D., Andolfo, G., Aiese Cigliano, R., Sanseverino, W., and Ercolano, M. R. (2022). PRGdb 4.0: an updated database dedicated to genes involved in plant disease resistance process. Nucleic Acids Res. 50:D1483–D1490.

Chen, C., Wu, Y., Li, J., Wang, X., Zeng, Z., Xu, J., Liu, Y., Feng, J., Chen, H., He, Y., et al. (2023). TBtools-II: A "one for all, all for one" bioinformatics platform for biological big-data mining. Mol. plant 16:1733–1742.

Chu, N., Zhou, J-R., Rott, P., Li, J., Fu, H-Y., Huang, M-T., Zhang, H-L., and Gao, S-J. (2022). *ScPR1* plays a positive role in the regulation of resistance to diverse stresses in sugarcane (*Saccharum* spp.) and *Arabidopsis thaliana*. Ind. Crop Prod. 180: 114736.

Kim, D., Langmead, B., and Salzberg, S. L. (2015). HISAT: a fast spliced aligner with low memory requirements. Nat. Methods 12:357–360.

Kosugi, S., Hasebe, M., Tomita, M., and Yanagawa, H. (2009). Systematic identification of cell cycle-dependent yeast nucleocytoplasmic shuttling proteins by prediction of composite motifs. Proc. Natl. Acad. Sci. U.S.A. 106:10171–10176.

Kumar, S., Stecher, G., Li, M., Knyaz, C., and Tamura, K. (2018). MEGA X: Molecular evolutionary genetics analysis across computing platforms. Mol. Biol. Evol. 35:1547–1549.

Li, Z., and Xing, D. (2011). Mechanistic study of mitochondria-dependent programmed cell death induced by aluminium phytotoxicity using fluorescence techniques. J. Exp. Bot. 62:331–343.

Ling, H., Fu, X., Huang, N., Zhong, Z., Su, W., Lin, W., Cui, H., and Que, Y. (2022). A sugarcane smut fungus effector simulates the host endogenous elicitor peptide to suppress plant immunity. New Phytol. 233:919–933.

Ling, H., Wu, Q., Guo, J., Xu, L., and Que, Y. (2014). Comprehensive selection of reference genes for gene expression normalization in sugarcane by real time quantitative rt-PCR. PLoS One 9:e97469.

Liu, K., Shi, L., Luo, H., Zhang, K., Liu, J., Qiu, S., Li, X., He, S., and Liu, Z. (2024). *Ralstonia solanacearum* effector *RipAK* suppresses homodimerization of the host transcription factor ERF098 to enhance susceptibility and the sensitivity of pepper plants to dehydration. Plant J. 117:121–144.

Livak, K. J., and Schmittgen, T. D. (2001). Analysis of relative gene expression data using real-time quantitative PCR and the 2^-ΔΔCT^ Method. Methods 25:402–408.

Love, M. I., Huber, W., and Anders, S. (2014). Moderated estimation of fold change and dispersion for RNA-seq data with DESeq2. Genome Biol. 15:550.

Lu, L., Zhang, J., Zheng, X., Xia, N., Diao, Z., Wang, X., Chen, Z., Tang, D., and Li, S. (2024). OsMPK12 positively regulates rice blast resistance via OsEDC4-mediated transcriptional regulation of immune-related genes. Plant Cell Environ. 47:3712–3731.

Pan, Q., Cui, B., Deng, F., Quan, J., Loake, G. J., and Shan, W. (2016). RTP1 encodes a novel endoplasmic reticulum (ER)-localized protein in *Arabidopsis* and negatively regulates resistance against biotrophic pathogens. New Phytol. 209:1641–1654.

Shang, L., He, W., Wang, T., Yang, Y., Xu, Q., Zhao, X., Yang, L., Zhang, H., Li, X., Lv, Y., et al. (2023). A complete assembly of the rice Nipponbare reference genome. Mol. Plant 16:1232–1236.

Sun, T., Chen, Y., Feng, A., Zou, W., Wang, D., Lin, P., Chen, Y., You, C., Que, Y., and Su, Y. (2023). The allene oxide synthase gene family in sugarcane and its involvement in disease resistance. Ind. Crop Prod. 192:116136.

Sun, T., Wu, Q., Zang, S., Zou, W., Wang, D., Wang, W., Shen, L., Zhang, S., Su, Y., and Que, Y. (2024). Molecular insights into OPR gene family in *Saccharum* identified a *ScOPR2* gene could enhance plant disease resistance. Plant J. 120:335–353.

Tian, F., Yang, D. C., Meng, Y. Q., Jin, J., and Gao, G. (2020). PlantRegMap: charting functional regulatory maps in plants. Nucleic Acids Res. 48:D1104–D1113.

Xu, D., Marquis, K., Pei, J., Fu, S. C., Cağatay, T., Grishin, N. V., and Chook, Y. M. (2015). LocNES: a computational tool for locating classical NESs in CRM1 cargo proteins. Bioinformatics 31:1357–1365.

Yang, A., Dai, X., and Zhang, W. H. (2012). A R2R3-type MYB gene, *OsMYB2*, is involved in salt, cold, and dehydration tolerance in rice. J. Exp. Bot. 63:2541–2556.

You, Q., Zhai, K., Yang, D., Yang, W., Wu, J., Liu, J., Pan, W., Wang, J., Zhu, X., Jian, Y., et al. (2016). An e3 ubiquitin ligase-bag protein module controls plant innate immunity and broad-spectrum disease resistance. Cell Host Microbe 20:758–769.

Zang, S., Qin, L., Zhao, Z., Zhang, J., Zou, W., Wang, D., Feng, A., Yang, S., Que, Y., and Su, Y. (2022). Characterization and functional implications of the nonexpressor of pathogenesis-related genes 1 (*NPR1*) in *Saccharum*. Int. J. Mol. Sci. 23:7984.

Zang, S., Wu, Q., Wang, D., Li, Z., Sun, T., Sun, X., Cui, T., Su, Y., Wang, H., and Que, Y. (2025). Cellular heterogeneity and immune responses to smut pathogen in sugarcane. Plant Biotechnol. J. 23:2608–2610.

Zhang, C., Li, Z., Sun, T., Zang, S., Wang, D., Su, Y., Wu, Q., and Que, Y. (2024). Sugarcane *ScCAX4* is a negative regulator of resistance to pathogen infection. J. Agric. Food Chem. 72:13205–13216.

Zhang, S., Xu, Z., Sun, H., Sun, L., Shaban, M., Yang, X., and Zhu, L. (2019). Genome-Wide Identification of Papain-Like Cysteine Proteases in *Gossypium hirsutum* and Functional Characterization in Response to *Verticillium dahliae*. Front Plant Sci, 10:134.

# Supplemental Figures


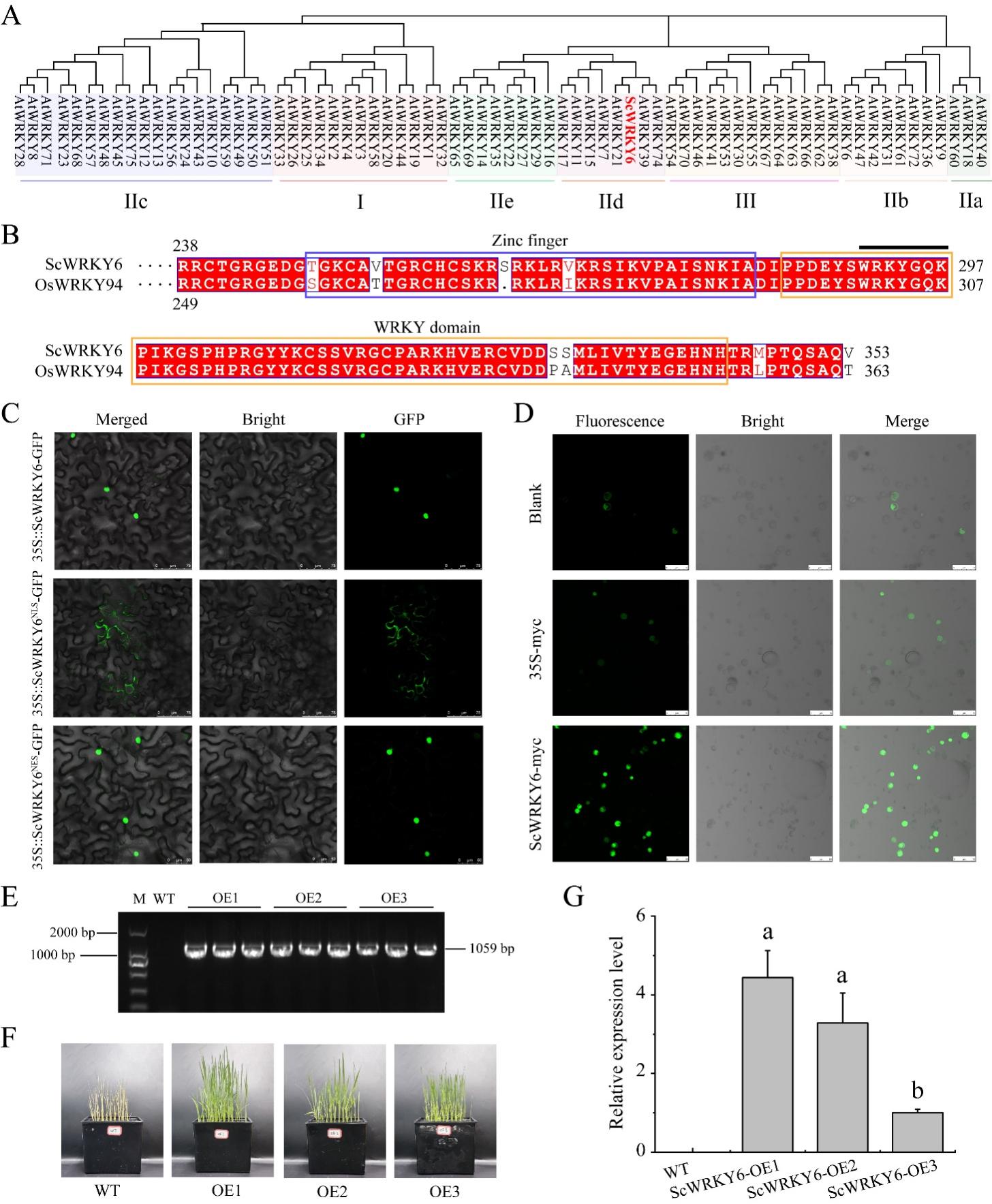


**Figure S1.** Characterization of ScWRKY6 in sugarcane and confirmation of transgenic rice overexpressing *ScWRKY6*. (A) Phylogenetic tree of the ScWRKY6 protein and *Arabidopsis* WRKY proteins. (B) Sequence alignment of ScWRKY6 and OsWRKY89 proteins. Blue boxes indicate WRKY domains, yellow boxes indicate zinc finger motifs, and black underlines mark the conserved WRKYGQK motif. (C) Subcellular localization of ScWRKY6^NLS^ and ScWRKY6^NES^ proteins in *N. benthamiana.* (D) Sugarcane protoplasts transiently transfected with ScWRKY6 and empty vector were stained with H₂DCF-DA to observe the accumulation of intracellular ROS. Blank, 35S-myc, and ScWRKY6-myc represented the blank control, vector control, and ScWRKY6 overexpression group, respectively. Bars=50 μm. (E) PCR detection of genomic DNA from T₃ generation transgenic rice plants overexpressing *ScWRKY6.* (F) Three T_3_ generation lines of ScWRKY6-OE plants selected through 0.05% glufosinate treatment. (G) Relative expression levels of *ScWRKY6* in transgenic and WT plants.


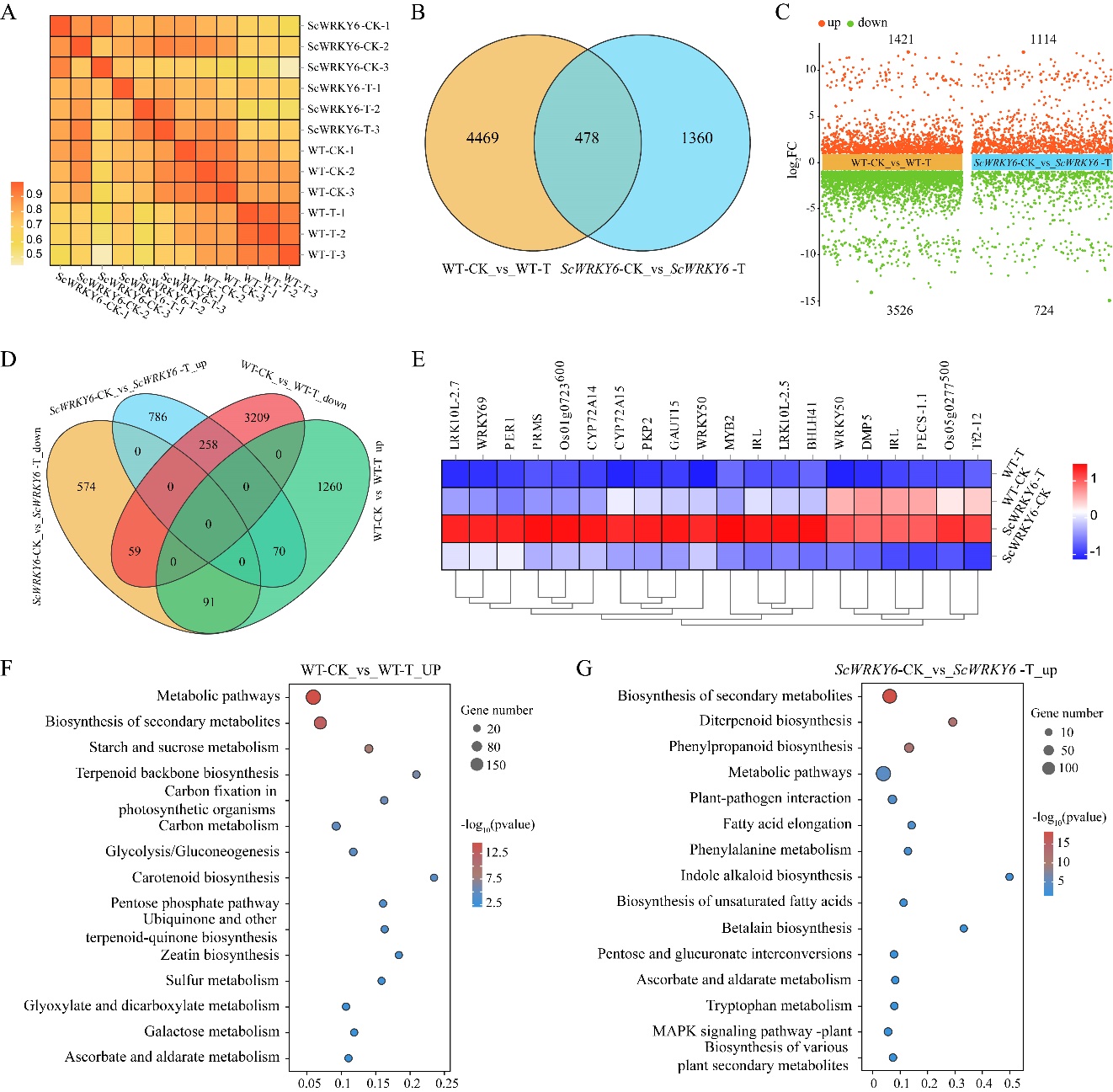


**Figure S2.** Transcriptome analysis of *ScWRKY6*-overexpressing transgenic lines and WT plants in response to disease infection. (A) Pearson correlation based on all expressed genes. *ScWRKY6*-CK, *ScWRKY6*-T, WT-CK, and WT-T represent *ScWRKY6*-OE and WT tobacco inoculated with *M. oryzae* for 0 d (CK) and 7 d (T), respectively. Each sample was set with three biological replicates. (B) Venn diagrams of DEGs between WT-CK_vs_WT-T and *ScWRKY6*-CK_vs_*ScWRKY6*-T. (C) The number of DEGs in WT-CK_vs_WT-T and *ScWRKY6*-CK_vs_*ScWRKY6*-T. (D) Venn diagrams of all genes exhibiting up- or down-regulated expression between WT-CK_vs_WT-T and *ScWRKY6*-CK_vs_*ScWRKY6*-T. (E) Expression profiles of genes down-regulated in WT-T and up-regulated in *ScWRKY6*-T after inoculation. (F) Top 15 KEGG pathway classification of the up-regulated genes in WT-CK_vs_WT-T. (G) Top 15 KEGG pathway classification of the up-regulated genes in *ScWRKY6*-CK_vs_*ScWRKY6*-T. WT-CK_vs_WT-T and ScWRKY6-CK_vs_ScWRKY6-T denote DEGs between CK and T after inoculation, positive log_2_ (fold change) indicates higher expression in T relative to CK.


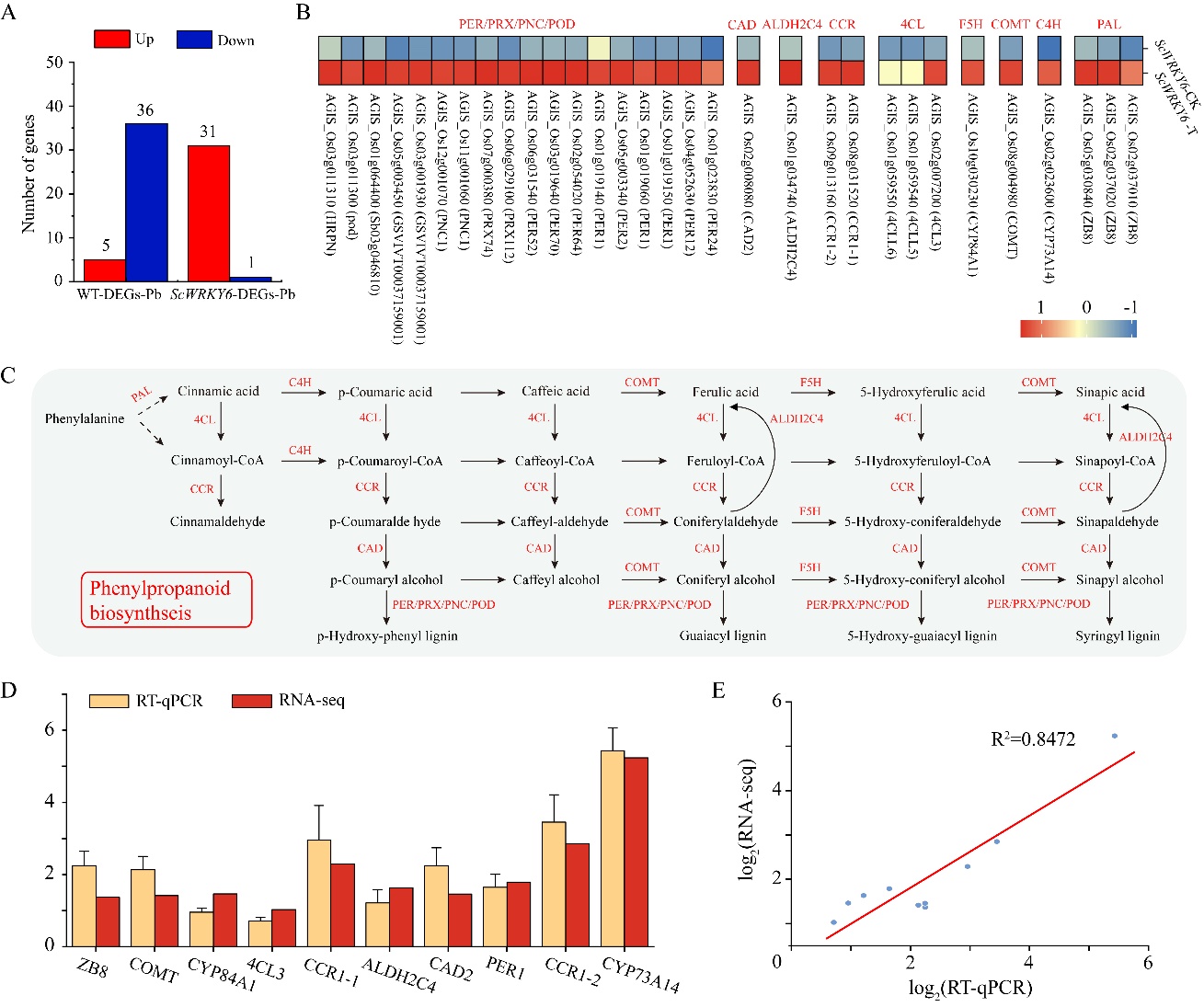


**Figure S3.** Overexpression of *ScWRKY6* in plants activated the phenylpropanoid biosynthesis pathway after inoculation with *M. oryzae*. (A) The number of DEGs between WT-CK_vs_WT-T and *ScWRKY6*-CK_vs_*ScWRKY6*-T in the phenylpropanoid biosynthesis pathway. (B) Heatmap diagrams of phenylpropanoid biosynthesis pathway genes in *ScWRKY6*-CK and *ScWRKY6*-T. (C) The phenylpropanoid biosynthesis pathways in *ScWRKY6*-CK_vs_*ScWRKY6*-T. The red fonts indicated up-regulated genes. (D) RT-qPCR verification of ten phenylpropanoid biosynthesis pathway genes. (E) Correlation of RT-qPCR (Log_2_ scale) and RNA-seq data (Log_2_ FC) of ten phenylpropanoid biosynthesis pathway genes.


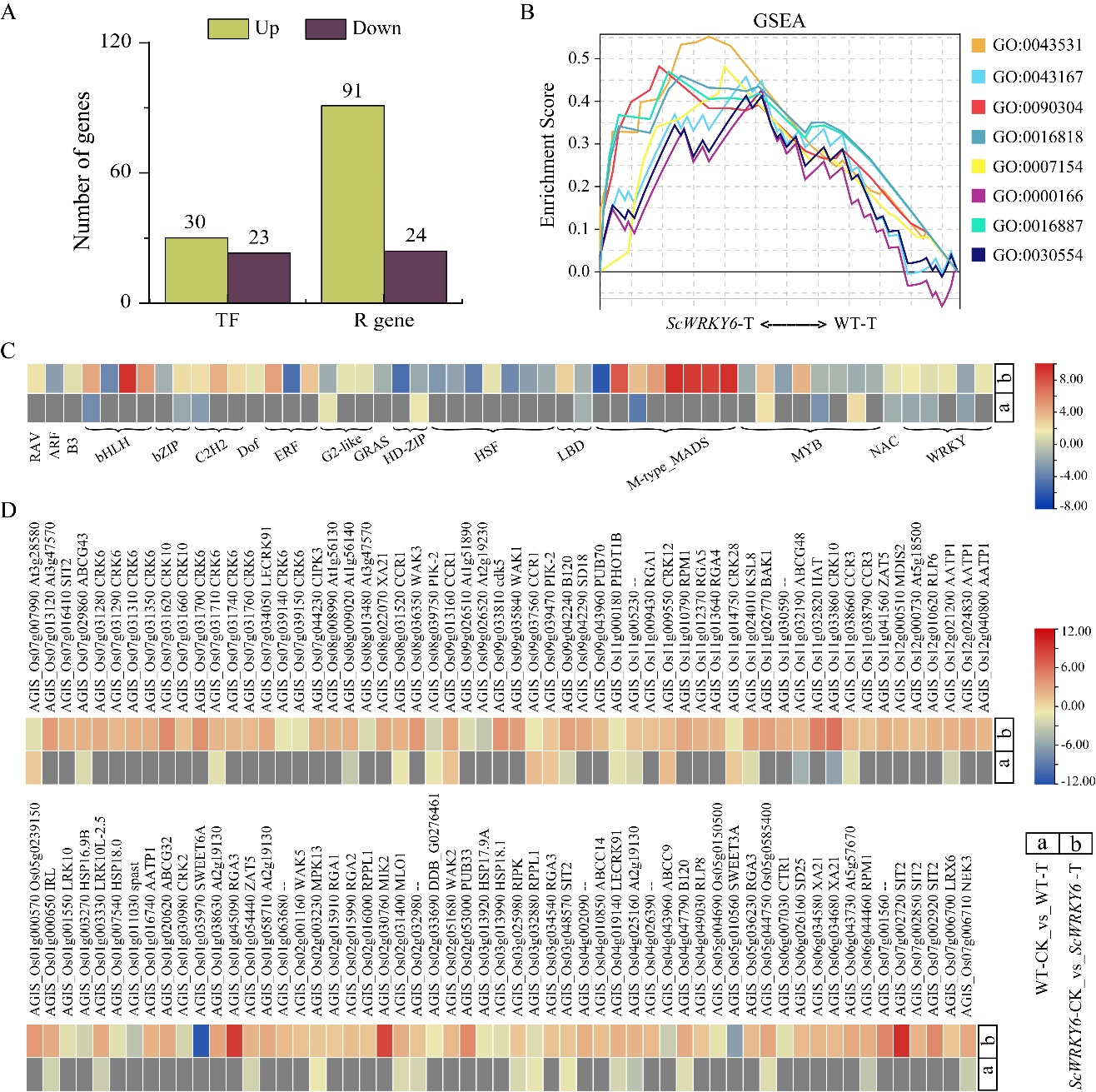


**Figure S4.** Transcription factors (TFs) and resistance (*R*) genes in *ScWRKY6*-OE related to disease resistance. (A) Numbers of TFs and *R* genes in the *ScWRKY6*-CK_vs_*ScWRKY6*-T. (B) GSEA analysis of 115 *R* genes in the *ScWRKY6*-T_vs_WT-T. (C, D) Heatmap diagrams of TFs and *R* genes in WT-CK_vs_WT-T and *ScWRKY6*-CK_vs_*ScWRKY6*-T. The gray box represented the genes with no expression. WT-CK_vs_WT-T and ScWRKY6-CK_vs_ScWRKY6-T denote DEGs between CK and T after inoculation, positive log_2_ (fold change) indicates higher expression in T relative to CK.


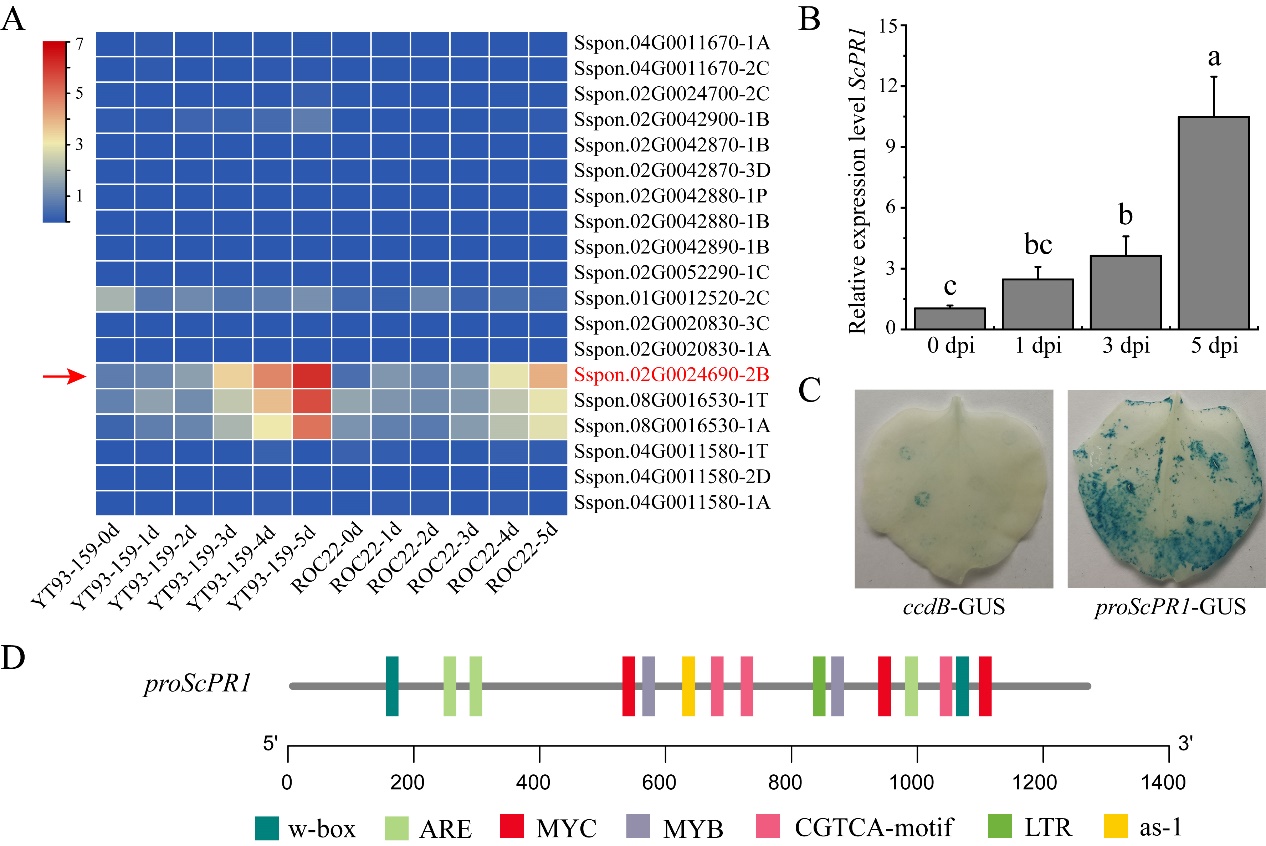


**Figure S5.** Characterization of the *ScPR1* gene and its promoter in sugarcane. (A) Expression patterns of 19 previously reported *SsPR1* genes in response to *S. scitanmineum* infection in the smut-susceptible variety ROC22 and the smut-resistant variety YC05-179. Red marker represents candidate gene. (B) Relative expression of *ScPR1* gene in sugarcane ROC22 after inoculation with *S. scitanmineum*. dpi: days post infection. (C) *pro-ScPR1* promoter transcriptional activities revealed by GUS staining. (D) Distribution and functional prediction of cis-acting regulatory elements of *ScPR1* gene promoter.


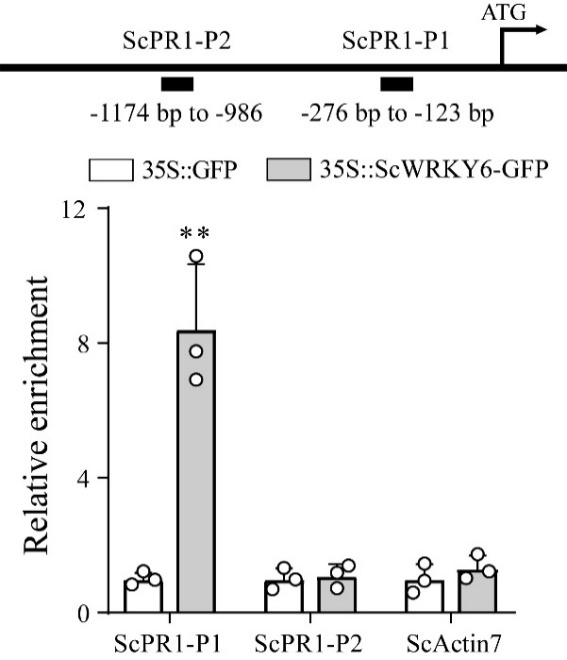


**Figure S6.** ChIP-qPCR assay in sugarcane protoplasts. 35S::ScWRKY6-GFP and 35S::GFP were transiently expressed, and chromatin was immunoprecipitated with anti-GFP magnetic beads. ChIP-qPCR with specific primers detected *ScPR1* promoter fragments in the eluates. The *ScActin7* promoter was used as an internal reference. Values represent enrichment relative to the input fraction (mean ± SD, n = 3). Statistical significance was determined by Student’s t-test (***p* < 0.01).


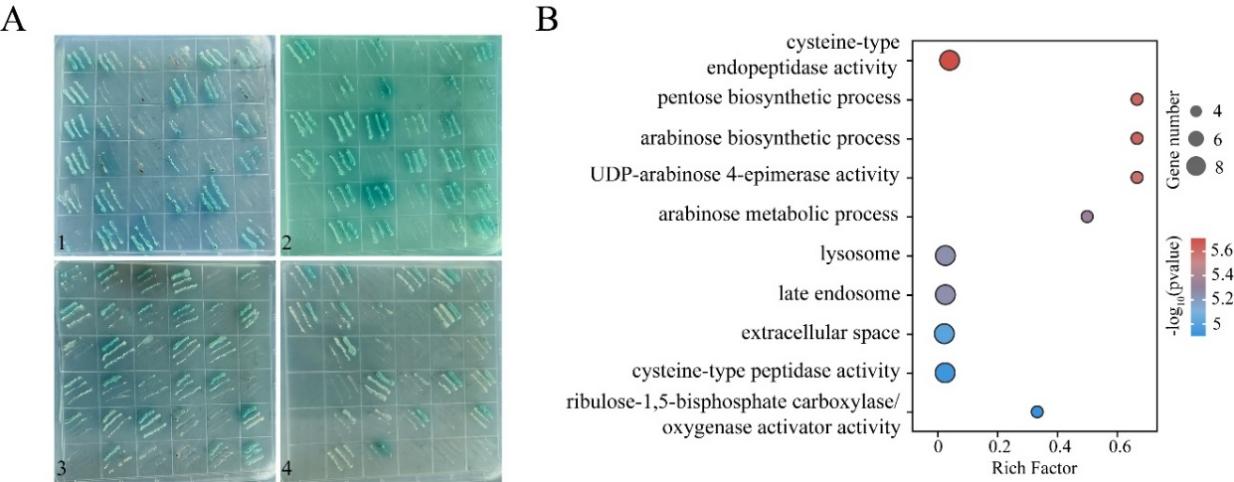


**Figure S7.** ScWRKY6 was used to screen a cDNA yeast library of the sugarcane smut pathogen and the interaction between ScSAG39 and ScWRKY6 was verified. (A) ScWRKY6 was used to screen a cDNA yeast library from the sugarcane smut pathogen-infected bud. Co-transformed cells were streaked on the selective medium SD/−Ade/−His/−Leu/−Trp + X-α-Gal. (B) GO functional annotations of the proteins interacting with ScWRKY6.


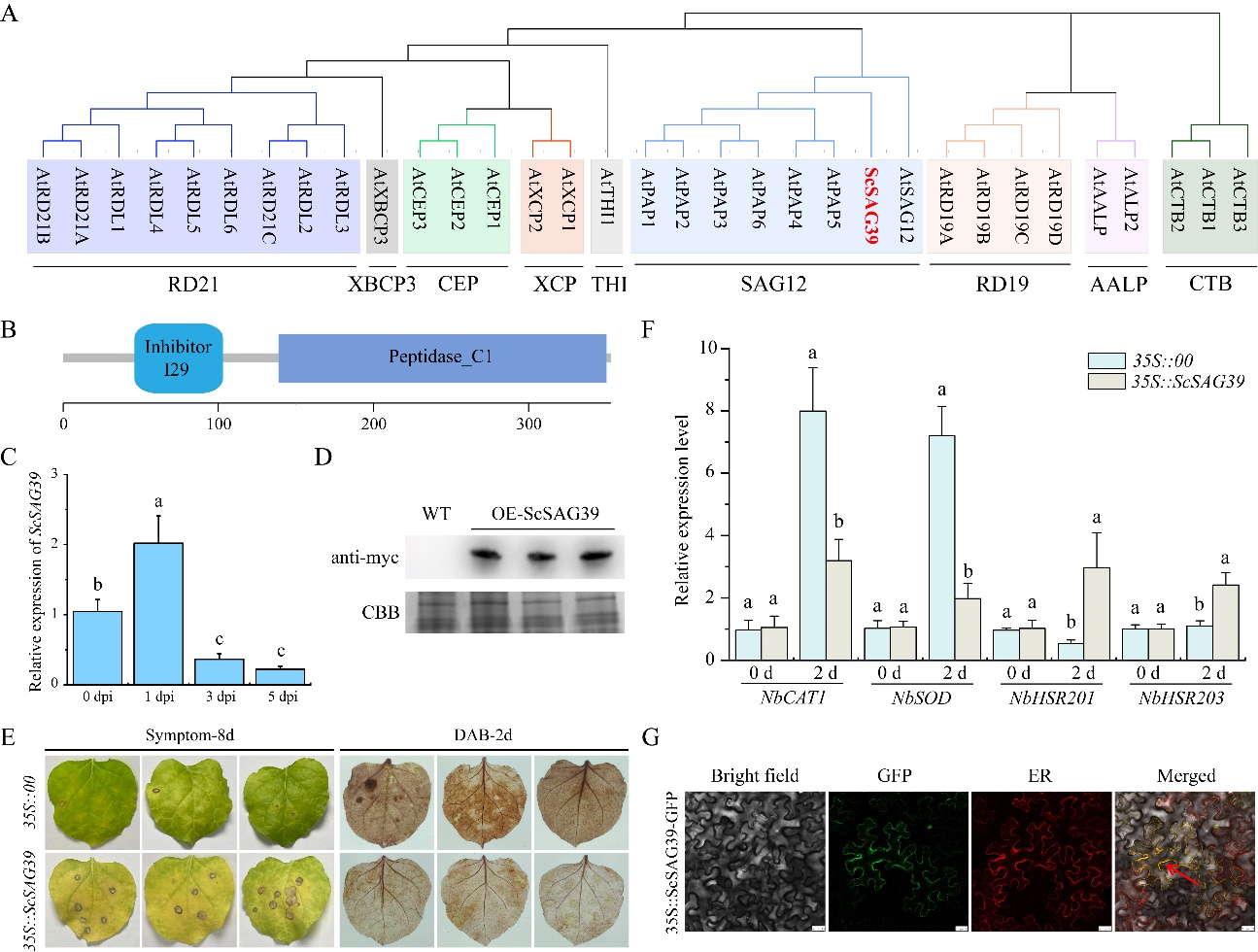


**Figure S8.** *ScSAG39* negatively regulated plant disease resistance. (A) Phylogenetic tree of the ScSAG39 protein and *Arabidopsis* PLCP proteins. (B) Conserved domains of ScSAG39 protein. (C) Relative expression levels of the *ScWRKY6* gene in sugarcane ROC22 after inoculation with *S. scitanmineum*. dpi: days post infection. (D) Immunoblot was performed using an anti-Myc antibody to detect ScSAG39 protein expression in WT and transiently overexpressing *ScSAG39* leaves. Coomassie brilliant blue (CBB) staining was performed to show that the proteins had been loaded equally. (E) The disease symptoms at 8 dpi and DAB staining at 2 dpi of *N*. *benthamiana* leaves inoculated with *Alternaria alternate*. dpi: days post infection. (F) Expression of immune-related marker genes in *N*. *benthamiana* leaves at 0 d and 2 d after inoculation with *A. alternate*. (G) Subcellular location of ScSAG39 proteins in *N. benthamiana*. ScSAG39 was co-localized with the marker ER-mCherry on the endoplasmic reticulum. Red arrows indicated endoplasmic reticulum membrane. Bar=25 μm.


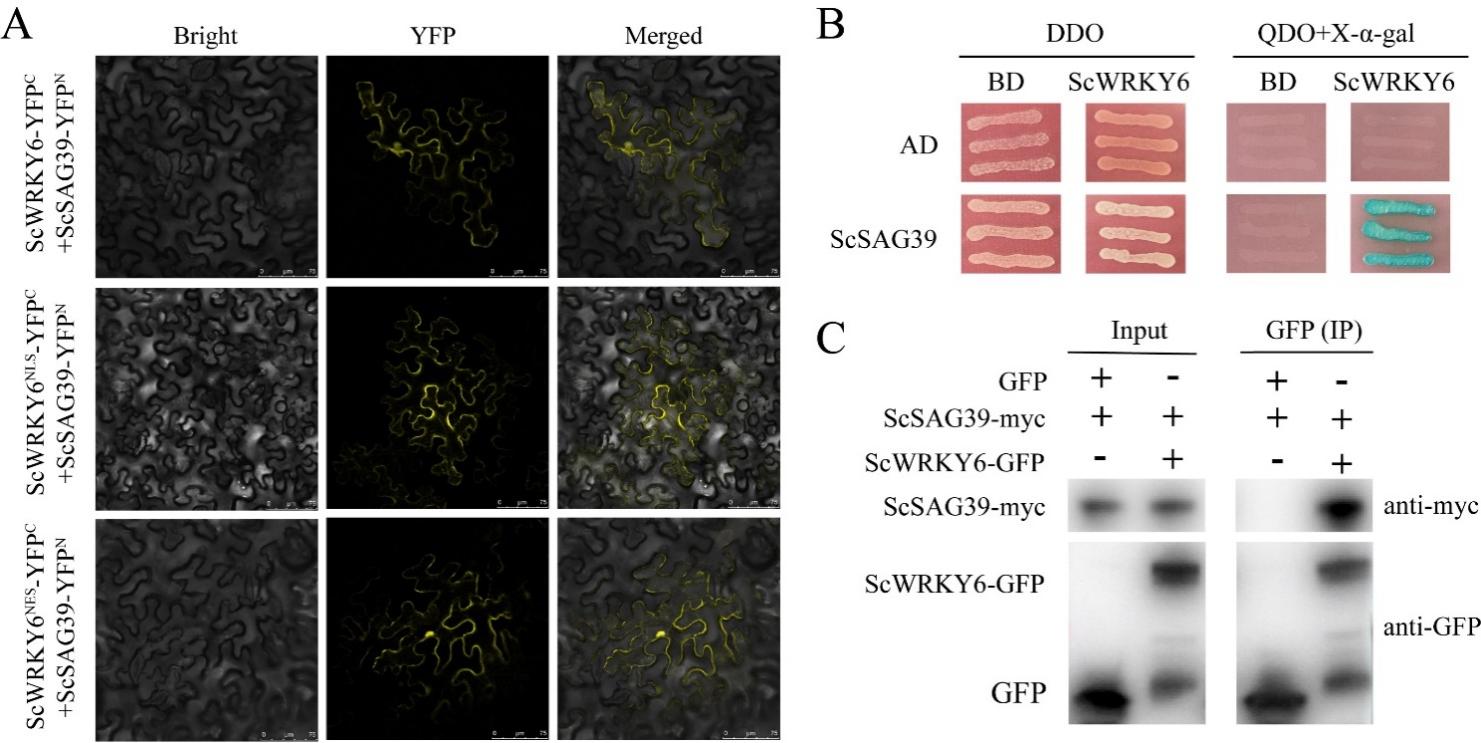


**Figure S9.** ScSAG39 directly interacted with ScWRKY6. (A) BiFC assays were performed in *N. benthamiana* to examine the interactions between ScWRKY6 and ScSAG39, ScWRKY6NLS and ScSAG39, as well as ScWRKY6NES and ScSAG39. ScSAG39, ScWRKY6, ScWRKY6^NLS^ and ScWRKY6^NES^ were fused to the N-terminus (YFP^N^) and C-terminus (YFP^C^) of YFP, respectively, and co-expressed in *N. benthamiana* leaves. (B) ScSAG39 interacted with ScWRKY6 in yeast. Yeast cells harboring ScSAG39-AD and ScWRKY6-BD could grow on both DDO (SD/-Leu/-Trp) and QDO (SD/- Leu/-Trp/-His/-Ade) + X-a-gal media, while the negative controls failed to grow. (C) Co-IP assay detecting interaction of ScSAG39 with ScWRKY6. The ScSAG39-myc fusion protein was co-expressed with ScWRKY6-GFP and GFP (used as a control) in *N. benthamiana* leaves. Immunoprecipitation was performed using anti-GFP beads, followed by immunoblotting with anti-GFP and anti-myc antibodies.


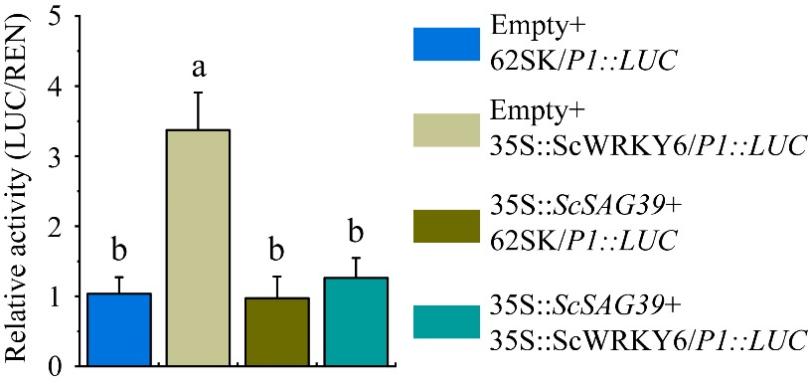


**Figure S10.** The expression of *ScSAG39* repressed the activity of the *ScPR1* promoter that was activated by ScWRKY6. The plasmids *proScPR1-P1::LUC* and 35S::ScWRKY6, with or without 35S::ScSAG39-GFP, were co-transfected into *N. benthamiana* leaves. A GFP-empty vector was used as a control. At 48 hours post-infiltration, the transfected leaves were harvested for luciferase imaging and measurement of relative LUC activity. Different letters indicate significance at *p* < 0.01.


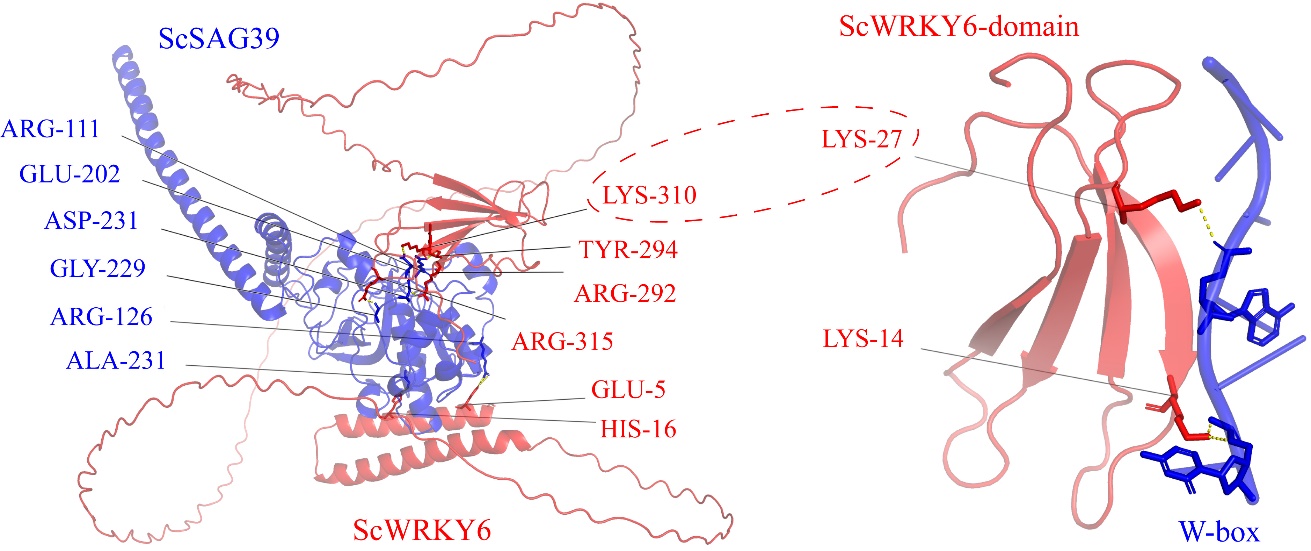


**Figure S11.** Molecular docking analysis of protein–protein and protein–DNA interactions involving ScWRKY6. Two molecular docking models were constructed using AlphaFold 3: one based on the full-length amino acid sequences of ScSAG39 and ScWRKY6, and the other based on the WRKY domain of ScWRKY6 and the W-box element. The ellipse markers indicate the overlapping binding sites. Molecular docking visualization was performed using PyMOL software.
